# Oncological safety of nipple-sparing mastectomy after neoadjuvant chemotherapy: a systematic review

**DOI:** 10.1590/0100-6991e-20233515-en

**Published:** 2023-07-26

**Authors:** LEONARDO NISSEN, ISABELA CHAVES MONTEIRO SOARES, RUBENS SILVEIRA DE LIMA, CICERO DE ANDRADE URBAN, IRIS RABINOVICH

**Affiliations:** 1 - Hospital de Clínicas da UFPR, Departamento de Tocoginecologia - Curitiba - PR - Brasil; 2 - Centro de Doeças da Mama - CDM Curitiba, Mastologia - Curitiba - PR - Brasil

**Keywords:** Neoadjuvant Therapy, Mastectomy, Subcutaneous, Breast Neoplasms, Terapia Neoadjuvante, Mastectomia Subcutânea, Neoplasias da Mama

## Abstract

**Background:**

the use of nipple-sparing mastectomy (NSM) in local advanced breast cancer after neoadjuvant chemotherapy (NQT) is increasing, despite few studies on the subject. The aim of this systematic review was to determine the safety of NSM after neoadjuvant chemotherapy.

**Methods:**

for this systematic review we searched MEDLINE; Cochrane; Scientific Electronic Library Online (SciELO); Embase and Scopus. A literature search of all original studies including randomized controlled trials, cohort studies and case-control studies comparing women undergoing NSM after neoadjuvant chemotherapy for breast cancer was undertaken. Outcomes were locoregional recurrence (LRR), nipple recurrence and distant recurrence (DR). Data analysis was undertaken to explore the safety of NSM after NQT. The quality of the evidence was assessed with the Cochrane risk of bias tool. This study is registered on PROSPERO, number CRD42021276778.

**Findings:**

a total of 437 articles were identified. Four articles were included with 1466 patients all of which had a high to serious risk of overall bias. Local recurrence in the NSM after the NQT group ranged from zero to 9.8%. Nippleareolar complex (NAC) recurrence ranged from zero to 2.1%. The distant recurrence rate ranged from 6.5% to 16%. Due to the lack of pattern among the control groups, it was not possible to perform a meta-analysis*.*

**Interpretation:**

*t*his review provides information for decision making in performing NSM after NQT. Despite the low rates of local recurrence and patients should be counseled about limited oncological information.

## RESUMO

**Introdução:**

O uso de mastectomia preservadora de complexo aréolo-papilar (MPCAP) no câncer de mama localmente avançado após quimioterapia neoadjuvante (QTN) é crescente, apesar de ainda haver poucos estudos abordando o assunto. O objetivo desta revisão sistemática foi determinar a segurança da MPCAP após a quimioterapia neoadjuvante.

**Métodos:**

para esta revisão sistemática, pesquisamos no MEDLINE; Cochrane; Scientific Electronic Library Online (SciELO); Embase e Scopus. Foi realizada uma busca na literatura de todos os estudos originais, incluindo ensaios clínicos randomizados, estudos de coorte e estudos de caso-controle comparando mulheres submetidas a MPCAP após quimioterapia neoadjuvante para câncer de mama. Os desfechos foram recorrência locorregional, recidiva em papila e recorrência à distância. A análise dos dados foi realizada para avaliar a segurança da mastectomia preservadora de complexo aréolo-papilar após o QTN. A qualidade da evidência foi avaliada com a ferramenta de avaliação de risco de viés da Cochrane - ROBINS-I. Este estudo está registrado no PROSPERO, número CRD42021276778.

**Resultados:**

Um total de 437 artigos foram identificados. Quatro artigos foram incluídos na análise, totalizando 1466 pacientes, todos com risco de viés geral moderado a alto. A recorrência local no grupo MPCAP após QTN variou de zero a 9,8%. A recorrência no complexo aréolo-papilar (CAP) variou de zero a 2,1%. A taxa de recorrência à distância variou de 6,5% a 16%. Devido à falta de padrão entre os grupos de controle, não foi possível realizar uma meta-análise.

**Interpretação:**

esta revisão fornece informações para a tomada de decisão na realização de NSM após QTN. Apesar das baixas taxas de recorrência local, os pacientes devem ser orientados sobre as informações oncológicas limitadas.

## INTRODUCTION

Breast cancer is the most common cancer in the world. According to the Global Cancer Observatory, there were an estimated 2.26 million new cases in 2020 and 684,996 deaths^1^.

Breast cancer treatment is constantly changing. Surgery is the main one, and its aesthetic results have been improving, even in advanced diseases, without compromising oncological safety. Breast-conserving surgery is the standard treatment nowadays; however, mastectomy is still required in some cases.

Radical mastectomies have been increasingly replaced by less aggressive procedures, which allow for better immediate breast reconstruction results. Skin-sparing mastectomy (SSM) was first described by Toth and Lappert and consists of removing the glandular tissue of the breast and the nipple-areola complex (NAC), preserving the skin^2^. Skin and NAC-sparing mastectomy, also known as nipple sparing mastectomy (NSM), preserves the cutaneous envelope and NAC, further improving aesthetic results and patients’ quality of life^3^.

NSM has been increasingly used, and its safety is well established in the treatment of early-stage breast cancer, as well as risk-reducing surgeries due to germline mutations. However, there are few studies that have evaluated the oncological safety of this technique in higher-risk patients, who are usually submitted to neoadjuvant chemotherapy^4^. Neoadjuvant chemotherapy (NQT) is currently a major pillar of breast cancer treatment, particularly in more aggressive molecular subtypes, such as triple negative and HER2-positive diseases, as well as locally advanced disease.

As in the situations described above there is a greater risk of local recurrence, and since NSM is a recent technique in the treatment of breast cancer, there are concerns, mainly regarding the remaining retroareolar tissue and the possibility of recurrence in the NAC. The aim of this study was to perform a systematic review of the oncological safety of NSM for patients treated with neoadjuvant chemotherapy and to compare it with other mastectomy techniques.

## METHODS

This review is reported in accordance with PRISMA (Preferred Reporting Items for Systematic Review and Meta-Analyses) standards5. We registered the protocol in PROSPERO (International Perspective Register of Systematic Reviews) under number CRD42021276778, available at https://www.crd.york.ac.uk/prospero/display_record.php?ID=CRD42021276778 .

### Studies and Participants

We included studies with levels of evidence 1-3 according to the Oxford Center for Evidence-Based Medicines (RCTs, cohort, and case-control studies)^6^. We did not include single-group cohorts in the analysis, but results were collected and presented separately. We excluded case reports, case series, expert opinion, or conference abstracts.

We included women undergoing NSM after NQT for invasive breast carcinoma. We excluded studies that did not determine the relapse rate exclusively for the NSM group after NQT, as well as studies that performed neoadjuvant hormone therapy. We did not restrict minimum follow-up time or language.

### I**nterventions and Comparators**

The intervention of interest was NSM, the comparator was SSM or total mastectomy after neoadjuvant chemotherapy. NSM consists of removing the glandular tissue with preservation of the cutaneous envelope and the NAC. SSM consists of the removal of the glandular tissue and the NAC, with preservation of the cutaneous envelope. Total mastectomy consists of removing glandular tissue, NAC, and the skin, without immediate breast reconstruction.

### Outcome Measures

The primary outcome was the local recurrence rate during the follow-up interval, including NAC recurrence. The secondary outcome was the distant recurrence rate during the follow-up interval.

### Research Methods

We searched the following electronic databases, with no defined start date, until September 2022: MEDLINE via PubMed; Cochrane Library (including Cochrane Database of Systematic Reviews, Cochrane Central Register of Controlled Trials); Online Scientific Electronic Library (SciELO); Embase; and Scopus. We also examined the references of included articles.

One of the authors (LN) conducted the search, using appropriate keywords in English and Boolean logical operations. The MEDLINE search strategy is shown in Table S1 (additional information). Queries have been translated into the appropriate syntax for other databases. There was no language and date limitation for the search.

**Table S1:** Search strategy for MEDLINE.

#1	Search: (breast neoplasm [ MeSH Terms]) AND (Surgery[ MeSH Subheading])
#2	Search: mastectomy [MeSH Terms]
#3	Search: (breast*[Title/Abstract]) AND ((surg *[Title/Abstract]) OR (reconstruct*[Title/Abstract])))
#4	Search: mastectom*[Title/Abstract]
#5	Search: #1 OR #2 OR #3 OR #4
#6	Search: ((nipple*[Title/Abstract])) OR (areola*[Title/Abstract]) OR (nac *[Title/Abstract])) AND ((spare*[Title/Abstract])) OR (sparing* [Title/Abstract]) OR (preserv *[Title/Abstract]) OR (reposition*[Title/Abstract]))
#7	Search: #5 AND #6
#8	Search: (nipples[ MeSH Terms]) AND (organ sparing treatment [ MeSH Terms])
#9	Search: #7 OR #8
#10	Search: neoadjuvant therapy[MeSH Terms]
#11	Search: (neoadjuvant [Title/Abstract] AND ((chemotherapy[Title/Abstract]) OR (chemotherapy*[Title/Abstract]) OR (treat*[Title/Abstract]) OR (therapy*[Title/Abstract]))
#12	Search: #10 OR #11
#13	Search: #9 AND #12

Selected studies were imported into Rayyan^®7^. Duplicate articles were excluded. The selected articles were included in two stages. In the first step, two authors (LN and IS) examined all titles and abstracts, and articles with discrepancies were resolved by consensus or proceeded to the next step. In the second stage, the articles were evaluated in full. After the second stage, a senior author (IR) analyzed all remaining discrepancies.

### Data extraction, collection, and management

Two researchers (LN and IS) were responsible for extracting data from the selected studies and entering them into an Excel^®^ 2011 database. Discrepancies were discussed with a senior author (IR).

### Risk of bias assessment

We used the ROBINS-I tool (Risk of Bias for Assessing Non-Randomized Trials of Interventions) to assess the risk of bias and quality of each eligible trial (Table 1)^8^.

**Table 1:** Methodological bias of included studies using ROBINS-I.

Author	1	2	3	4	5	6	7	General
Santoro et al.^3^	Critical	Critical	Critical	Moderate	Low	Moderate	Moderate	Critical
Ryu et al.^9^	Moderate	Moderate	Moderate	Moderate	Low	Serious	Moderate	Critical
Agresti et al.^11^	Moderate	Moderate	Moderate	Low	Low	Moderate	Low	Moderate
Wu et al.^10^	Moderate	Moderate	Moderate	Moderate	Low	Low	Moderate	Moderate

1: Confusion bias; 2: Bias due to participant selection; 3: Bias in the classification of interventions; 4: Bias due to deviations from intended interventions; 5: Bias due to lack of data; 6: Bias in the measurement of results; 7: Bias in the reported result.

## RESULTS

**Figure 1:**
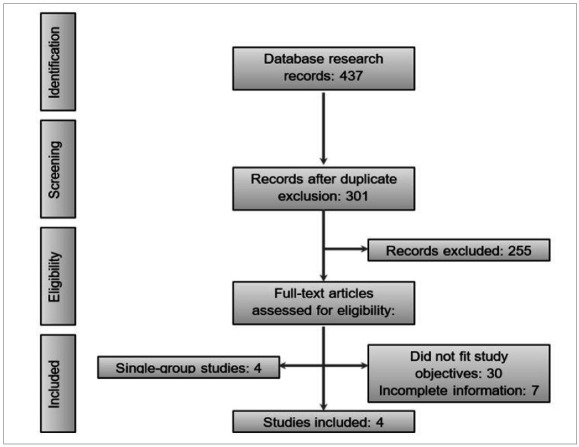
PRISMA flowchart for selection of studies.

We identified 437 studies across all search platforms and, after removing duplicate articles, 301 remained. After applying the eligibility criteria, were included four articles in the analysis of results (Figure 1).

The four articles were of level 3 evidence, and included 1,466 patients, of whom 466 were in study groups; however, only 312 were classified as of interest for this study. One hundred and fifty-four patients had been included in the study groups without having undergone NSM mastectomy and were excluded from the analysis. A total of 1,055 participants underwent SSM, upfront NSM, or conventional mastectomy as a control group. The follow-up time ranged from 35 to 68 months. Table 2 shows the summary of the included studies.

**Table 2:** Summary of included studies.

Autor	Year	Country	Type of study and period	Study group (n)	Control group (n)	Control intervention	Propensity Score Matching
Santoro et al.^3^	2015	Italy	Retrospective Jan/15-Jan/17	51	135	Upfront NSM	-
Ryu et al.^9^	2017	Korea	Retrospective Jan/08-Jun/15	13/18*	85	TM after NQT	-
Agresti et al.^11^	2016	Italy	Prospective cohort Jan/09-May/13	61	361/ 151	Upfront NSM/ TM after NQT	1:1 SG x TM after NQT 1:1** SG x NSM upfront 1:3*** SG x NSM upfront
Wu et al.^10^	2020	Korea	Retrospective Jan/10-Nov/16	187/136^&^	323	TM after NQT	1:1

NSM: skin and nipple-areolar complex sparing mastectomy; TM: total mastectomy; NQT: neoadjuvant chemotherapy; Upfront: surgery before neoadjuvant chemotherapy; SG: study group. *The study group included 13 patients undergoing NSM and 18 patients undergoing SSM after neoadjuvant chemotherapy. **balancing of the two groups in clinical and/or radiological tumor size at diagnosis. ***balancing the two groups with pathological tumor size. &: The study group included 187 patients undergoing NSM and 136 patients undergoing SSM after neoadjuvant chemotherapy.

Table 3 shows the oncological results. Local recurrence in the NSM group after NQT ranged from zero to 9.8%. Recurrence in NAC ranged from zero to 2.1%. The distant recurrence rate ranged from 6.5% to 16% in the study groups and from 7% to 28.2% in the control groups.

**Table 3:** Oncological results.

	Locoregional recurrence			Recurrence at a distance				Follow-up	
Author	NSM n (%)	GC n (%)	p	NSM n (%)	GC n (%)	p	NSM (months)	GC (months)
Santoro et al.^3^	3 (6%) / NACR 0	0	<0.01	6 (12%)	10 (7%)	0.3	35	35
Ryu et al.^9^	0	6 (7.1%)	n.r.	2 (6.5%)	24 (28.2%)	n.r.	38.2	45.8
Agresti et al.^11^	6 (9.8%) / NACR 1 (1.6%)	upfront NSM 10 (2.8%) NACR 0 / TM-PQ 16 (10.6%)	^&^p=0.655 ^✢^p=0.739 ^§^p=0.035	n.r.	n.r.	n.r.	46	42.5 (NSM upfront) 49.5 (TM-PQ)
Wu et al.^10^	LR 9 (4.8%) RR 13 (7%) / NACR 4 (2.1%)	RL11 (3.4%) RR 17 (5.3%)	n.r.	30 (16%)	60 (18.6%)	n.r.	67*	68

NSM: skin-sparing mastectomy and nipple-areolar complex; NAC: papillary areolar complex; CG: control group; TM-PQ: total mastectomy after neoadjuvant chemotherapy; LR: local recurrence; RR: regional recurrence; n.r.: not reported; NACR: recurrence in NAC. *Includes SSM with NSM. & SG x TM after NQT. ^*✢*^ SG x upfront NSM (pre NQT pairing). § SG x upfront NSM (post NQT pairing). Upfront: surgery before neoadjuvant chemotherapy.

Four single cohort articles had data collected separately, for simple description (Table 4). In these studies, the maximum local recurrence was 12%. NAC recurrence ranged from zero to 1.9%.

**Table 4:** Oncological results in single cohort articles.

Author	NSM after NQT	Follow up (months)	Locoregional recurrence	Recurrence in NAC	Recurrence at a distance
Jadeja et al.^15^	39	67.2	2 (5.1%)	0	5 (12.8%)
Galimberti et al.^16^	121	94	6 (5%)	1 (0.8%)	n.r.
Wu et al.^17^	319	63	38 (11.9%)	6 (1.9%)	7 (18.4%)
Wu et al.^18^	370	63	73 (12%)	7 (1.9%)	99 (16.3%)

NSM: nipple-sparing mastectomy; NAC: papillary areolar complex; NQT: neoadjuvant chemotherapy; n.r.: not reported.

## DISCUSSION

We found fewer than expected studies on NSM after NQT and we did not identify other reviews on the subject. Only four studies clearly highlighted its oncological outcomes and compared it with other techniques. Due to the lack of pattern between the control groups, we could not perform a meta-analysis.

Exclusively in participants submitted to NSM after NQT, locoregional recurrence ranged from zero to 11.8%, and recurrence in NAC, from zero to 2.1%. The total number of participants was 312, varying between 13 and 187 between studies. The follow-up time ranged from 35 to 68 months. The distant recurrence rate ranged from 6.5% to 16%.

The maximum local recurrence in the control groups was 10.6%, but the control group did not show a homogeneous intervention between studies, not allowing a meta-analysis. The total number of participants was 1,055, ranging from 85 to 512 participants. The distant recurrence rate ranged from 7% to 28.2% in the control groups.

In single-cohort studies, follow-up time ranged from 63 to 94 months. A total of 849 participants underwent NSM after NQT in these studies. The local recurrence rate ranged from 5% to 12%, with a recurrence rate in the NAC between zero and 1.9%. Distant recurrence ranged from 12.8% to 18.3%. Single cohort studies were not included in the analysis, however, due to the few studies conducted on the subject, we describe the findings separately to make this review more comprehensive.

Ryu et al.^9^ and Wu et al.^10^ included NSM and SSM in the study group for analysis but presented relapse rate data separately for the post-NQT NSM group. However, Santoro et al.^3^ showed a difference in the locoregional recurrence rate when comparing NSM after NQT and SSM after NQT (6% vs. 0%, p<0.01) and showed no difference in the systemic recurrence rate (12% vs. 0.7%, p=0.3) and mortality (4% vs. 2%, p=0.1).

Agresti et al.^11^ performed propensity score matching to obtain balanced subgroups in many observed covariates. Three subgroups were created to assess local disease-free survival (LDFS):

- Group 1 included post-NQT NSM in the study group and post-NQT total mastectomy (post-NQT TM) to assess locoregional recurrence. The 4-year LDFS of the post-NQT NSM and post-NQT TM cohorts was 0.89 (95% CI 0.77-0.95) and 0.93 (95% CI 0.83-0.97), respectively (HR = 1.31, 95% CI 0.40-4.35), the difference not being significant (p=0.655).
- Group 2 included post-NQT NSM in the study group and NSM (without NQT) to assess the role of tumor size before NQT in locoregional recurrence (tumor size before NQT as an equilibrium covariate). The risks of local recurrence were comparable between the two matched groups (HR = 1.23, 95% CI 0.37-4.04, p=0.739).
- Group 3 included post-NQT NSM in the study group and NSM (without NQT), to assess the role of tumor size after NQT in locoregional recurrence (tumor size after NQT as an equilibrium covariate). The size of the pathologic tumor after NQT was taken as the basis for comparing local recurrence, there being a significant difference: the 4-year LDFS was 0.89 (95% CI 0.77-0.9 5) in the group NSM post-NQT and 0.98 (95% CI 0.94-0.99) in the NSM (HR = 3.60, 95% CI 1.10-11.80, p=0.035).

Agresti et al.^11^ conclude that in patients undergoing chemotherapy, the risk of local recurrences after NSM is significantly associated with the stage of breast cancer at diagnosis before chemotherapy. It is not associated with the type of surgical procedure.

Locoregional recurrence showed a significant difference in the study by Santoro et al.^3^. However, the study group is composed of patients with more advanced clinical stages (stages II and III correspond to 96% in the study group and only 50% in the control group - SSM after NQT). Agresti et al.^11^ paired groups with propensity score matching and in pairing 1, which compared post-NQT NSM and post-NQT TM, there was no significant difference. Finally, in the NSM group (no NQT in the pairing 3 group), the local recurrence rate was significantly higher in patients with T2-T3 than T1 (0.8% and 6.3%, respectively, p=0.050). This data agrees with McBain et al.^12^, who have demonstrated a higher rate of local recurrence in more advanced tumors, as well as in younger patients, depending on the incision margins.

The study that showed a significant difference in local recurrence was from Santoro et al.^3^. However, the characteristics of participants in the study and control groups were different, with a higher clinical stage, a higher incidence of lymph node positive, and a higher incidence of HER2 positive or triple negative breast cancer in the study group. Another study, by Agresti et al.^11^, with a significant difference in local recurrence, also compared two distinct groups (pairing size in NSM post-NQT in the study group and NSM - without NQT). The other studies did not perform statistics, specifying only the NSM technique after NQT. However, in general, local recurrence rates were low (zero to 11.8%). Relapse rates in NAC were also low, not exceeding 2.1% in any of the evaluated studies.

A systematic review and meta-analysis by Sun et al.^13^ compared local and locoregional recurrence in the post-NQT setting between mastectomy and breast-conserving surgery (BCS). Patients with good NQT response showed no significant difference in local recurrence (LR), suggesting no difference in regional recurrence (RR) (OR=0.83.95%, CI 0.60-1.15, p=0. 26 and OR=0.56, 95%CI 0.33-0.93, p=0.03). Mean follow-up time ranged from 30 to 91 months. LR and RR had overall rates of 7.37% and 5.89% for mastectomies and 6.4% and 3.05% for BCS. Despite not specifying the type of mastectomy, the numbers are consistent with data from our review.

Acea-Nebril et al.^14^, in a recent publication, did not demonstrate a significant difference in oncological outcomes related to locoregional and distance recurrence between three groups of patients who underwent mastectomy and immediate reconstruction for breast carcinoma, with the study group consisting of patients who underwent NQT, and the two control groups, formed by patients who underwent systemic treatment after surgery and patients who did not need systemic treatment, respectively. Although lacking a longer follow-up to encourage decision-making, that work demonstrates the oncological safety trend, verified in our review and in the other studies presented. In addition, another important pillar addressed by the authors concerns postoperative complications in the study group. Systemic treatment can affect the rates of postoperative complications due to cicatricial and circulatory changes resulting from the cytostatic effect of the chemotherapy drugs used^15^. The authors demonstrated lower rates of implant loss in the study group (3.4%) compared with the control group that underwent chemotherapy after surgery and immediate reconstruction (13.2%).

Factors that impact relapse rates, such as the rate of radiotherapy, response to chemotherapy, and tumor subtypes, need to be better evaluated. This would allow individualization and improvement in decision-making regarding the patient’s treatment.

This review provides information for decision-making in performing NSM after NQT. As far as we know, this is the first review on this topic. Both tumor size and initial staging appear to be associated with higher rates of local recurrence in the NQT setting. However, neither local nor systemic recurrence appear to be related to surgical technique. NSM after NQT is an acceptable procedure in this setting despite limited data. Patients should be advised of limited oncological information.
